# Estimating and comparing the duration of adolescent growth peak in skeletal class I and III subjects using cervical vertebral maturation method

**DOI:** 10.1186/s40510-022-00420-9

**Published:** 2022-07-31

**Authors:** Mohammad H. Naderi, Mina Biria, Soheil Shahbazi, Sina Kousha, Kazem Dalaie, Mohammad Behnaz

**Affiliations:** 1 Research Institute for Dental Sciences, School of Dentistry, Shahid Beheshti University of Medical Sciences, Daneshjoo Blvd., Evin City, Tehran, Iran; 2 Department of Pediatric Dentistry, School of Dentistry, Shahid Beheshti University of Medical Sciences, Daneshjoo Blvd., Evin City, Tehran, Iran; 3 Department of Orthodontics, School of Dentistry, Shahid Beheshti University of Medical Sciences, Daneshjoo Blvd., Evin City, Tehran, Iran

**Keywords:** Cervical vertebrae, Adolescent, Age of onset, Cephalometry

## Abstract

**Background:**

Estimating skeletal maturation and growth potential is essential for developing adolescents' best orthodontic treatment plan. The purpose of this study was to compare the duration of adolescent growth peak in subjects of skeletal classes I and III using the cervical vertebral maturation (CVM) method.

**Methods:**

This retrospective cross-sectional study included 116 Iranian subjects (skeletal class I = 68, skeletal class III = 48) aged 8–16 years old and without previous orthodontic treatments. Using Steiner and Wits analyses, two independent examiners traced pre-treatment lateral cephalograms to determine the subjects' skeletal relationship. The skeletal maturation was then assessed using Baccetti's CVM method. The onset and duration of adolescent growth peak (interval of CS3–CS4) were compared between two skeletal classes and two genders using independent samples *t* test.

**Results:**

In skeletal class I and III subjects, the adolescent peak had a mean duration of 1.62 (± 1.33) and 2.00 (± 1.27) years, respectively. The average difference of 0.38 years (4.6 months) between the two groups was statistically significant (*p* < 0.001). Furthermore, the onset age of adolescent growth peak was 11.91 (± 1.32) and 12.08 (± 1.31) years old in class I and III subjects, respectively. This age difference was not statistically significant (*p* = 0.630). Males’ adolescent growth peak occurred 1.44 years later (*p* < 0.001) and lasted 0.20 years less (*p* < 0.001).

**Conclusions:**

The adolescent growth peak started at a similar age in class I and III subjects, but the latter experienced the peak for 4.6 months longer. Moreover, females had an earlier and more extended adolescent growth peak.

## Background

A human with regular development experiences periods of growth acceleration and deceleration [1]. Thus, estimating the degree of development and skeletal maturation is critical in determining the best treatment plan during adolescence [2]. In other words, starting the orthodontic treatment at the right time is as important as choosing the right treatment plan to achieve the least unwanted effects and the most stable outcomes [3]. As maxillofacial bones grow rapidly during the adolescent growth peak, growth modification treatments will produce the desired results if performed in this period [3].

Multiple factors influence the growth peak's onset and duration, including gender, ethnic background, nutritional habits, genetics, environment, and lifestyle [4, 5]. As a result, two patients of the same chronological age may be in two different skeletal maturation stages, making chronological age an inaccurate indicator to predict skeletal maturation [6]. Several biological indicators, such as increases in body height or weight, sexual maturation characteristics, dental development, and skeletal development, can be used alternatively to determine a patient's skeletal maturation and, thus, growth potential [7–9].

Two common ways of estimating skeletal development are hand-wrist radiographs and the Cervical Vertebral Maturation (CVM) method. In the CVM method, subjects will be classified as Cervical Stage 1 (CS1) to Cervical Stage 6 (CS6) based on the morphology of their cervical vertebrae, which changes during skeletal maturation [3]. The maximum mandibular growth has been recorded between CS3 and CS4; thus, the interval of CS3 and CS4 is regarded as the period of adolescent growth peak [2, 3]. In 1972, Lamparski introduced the primary standards of the CVM method as they relate to chronological age and skeletal maturation via hand-wrist maturation [10]. Later, in 2005, Baccetti et al. investigated the relationship between vertical growth of the mandibular ramus (as measured by Co-Go in lateral cephalograms) and the morphology of the second, third, and fourth cervical vertebrae (C2–C3–C4). As a result, an improved version of the CVM method was represented, which relied on three cervical vertebrae rather than Lamparski's method relying on the second through sixth vertebrae [3].

There are diverse opinions about the CVM method in the current literature. Although there are studies that have doubted the correlation between the CVM method and skeletal maturation [11–13], several studies have supported the CVM method as a reliable approach that can even be used instead of hand-wrist radiographs to assess stages of adolescent growth peak [14–16]. The CVM method has advantages such as the straightforward judgment of vertebral morphology and no need for additional X-ray exposure [1].

The orthodontic and orthopedic treatment plan varies in patients of different skeletal classes. Skeletal class III relationship has a multifactorial etiology, making its management challenging for orthodontists, despite the availability of various orthodontic, orthopedic, and surgical treatment modalities [17]. Furthermore, a few studies have reported that patients with skeletal class III relationship experience a longer adolescent growth peak than class I patients, which may be linked to their greater mandibular growth [18–20].

Due to the low number of studies comparing the adolescent growth peak between skeletal classes among the Iranian population, the purpose of this study was to assess the onset and duration of the mentioned peak in subjects of skeletal class I and III using the CVM method at Shahid Beheshti School of Dentistry, Tehran, Iran.

## Material and methods

In this retrospective cross-sectional study, 897 digital lateral cephalograms from the archives of Shahid Beheshti Dental School, Tehran, Iran, were analyzed in 2020–2021. The local ethics committee approved the current study. Sample size calculation was done using the formula below with a confidence level of 95% and a power of 80%. The mean and standard deviation parameters were extracted from the study of Jeelani et al. for each group [21]. The minimum required sample size for skeletal class I and class III groups was calculated to be 68 (female = 34, male = 34) and 40 (female = 20, male = 20), respectively. However, more subjects were allocated to the class III group (*n* = 48).

$$n = \frac{{(\delta_{1}^{ 2} + \delta_{2}^{ 2} ).\left( {Z_{1 - \alpha /2} + Z_{1 - \beta } } \right) ^{2} }}{{\left( {M_{1} - M_{2} } \right) ^{2} }}$$

The inclusion criteria of subjects were:

- Lateral cephalograms with proper quality and traceable C2, C3, and C4 vertebrae
- Age of 8–16
- Iranian ethnicity
- No previous orthodontic treatments
- No congenital missing of teeth or extractions
- No systemic diseases affecting body development
- CVM stage of CS3 or CS4

Two expert and calibrated examiners (one dentist and one pediatric dentistry resident) manually evaluated the CVM stage and skeletal classification for each cephalogram using Baccetti’s method and Steiner’s cephalometric analysis, respectively [3, 22]. Subjects with ANB = 2 ± 2° were considered skeletal class I, and subjects with ANB < 0° were considered skeletal class III. Wits analysis was also used in cases where ANB angle would be potentially erroneous. In this situation, two lines perpendicular to *A* and *B* points were drawn, intersecting the occlusal plane at *A*′ and *B*′. In class I and III subjects, the *A*′–*B*′ distance was 1 ± 1 mm and < − 2 mm, respectively [23]. Each examiner traced all cephalograms twice, one week apart, in random order (accordingly, each cephalogram was traced four times). Any disagreement was resolved by inter-examiner discussion or consulting an orthodontist. Dentitions were hidden on cephalograms, and examiners were blind to the subject’s age.

**Fig. 1:**
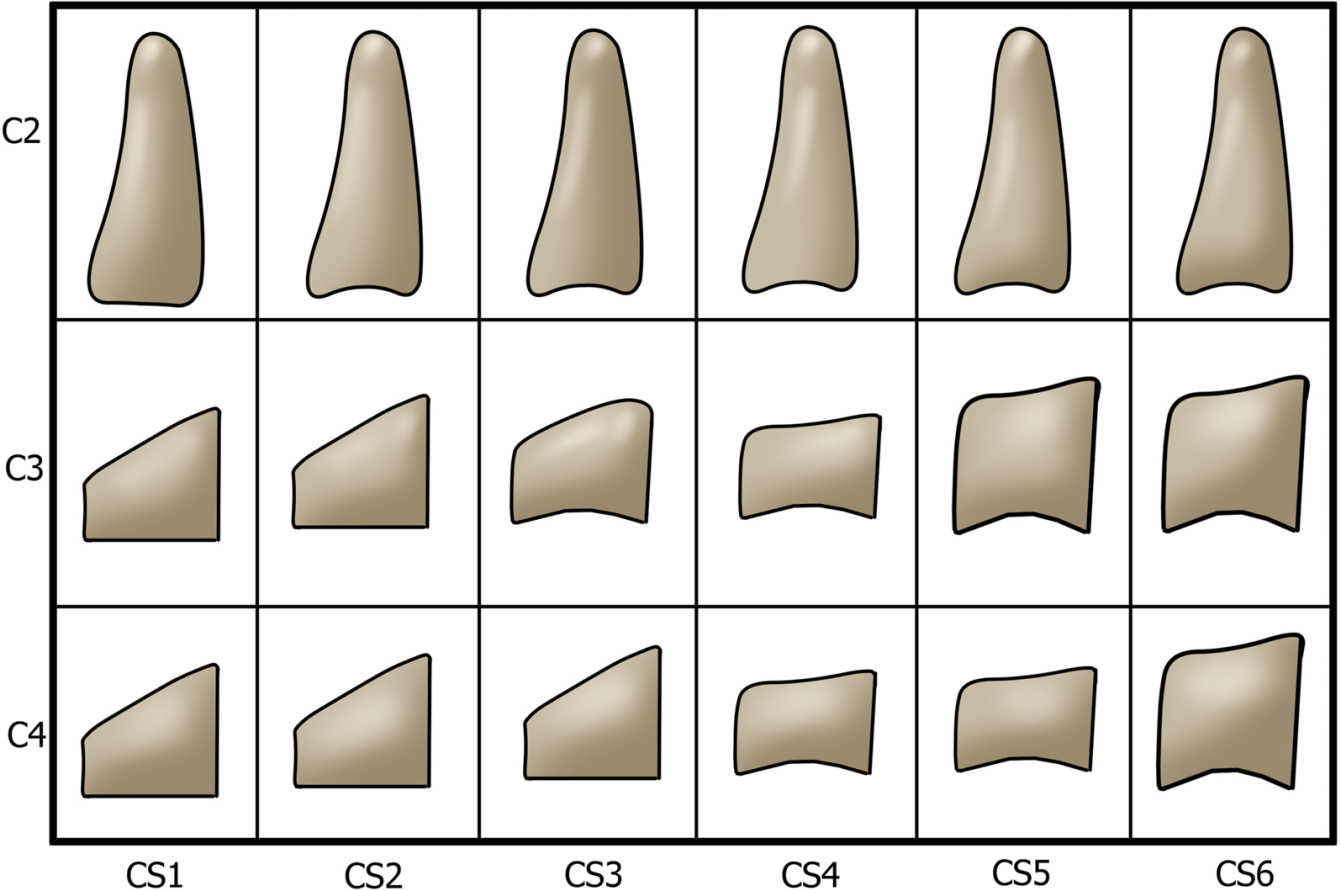
Cervical vertebral maturation staging, introduced by Baccetti. CS1: three vertebrae have flat inferior borders, and C3 and C4 are trapezoids in shape. CS2: the inferior border of C2 is concaved. C3 and C4 are still trapezoidal. CS3: the inferior borders of C2 and C3 are concaved. The shape of C3 and C4 may be a trapezoid or horizontal rectangle. CS4: concavity is visible in the inferior border of three vertebrae. C3 and C4 are in the shape of a horizontal rectangle. CS5: concavity is visible in the inferior border of three vertebrae. C3 and C4 may be shaped like a square or a horizontal rectangle. CS6: concavity is visible in the inferior border of three vertebrae. C3 and C4 may be in a shape of a square or vertical rectangle

**Fig. 2:**
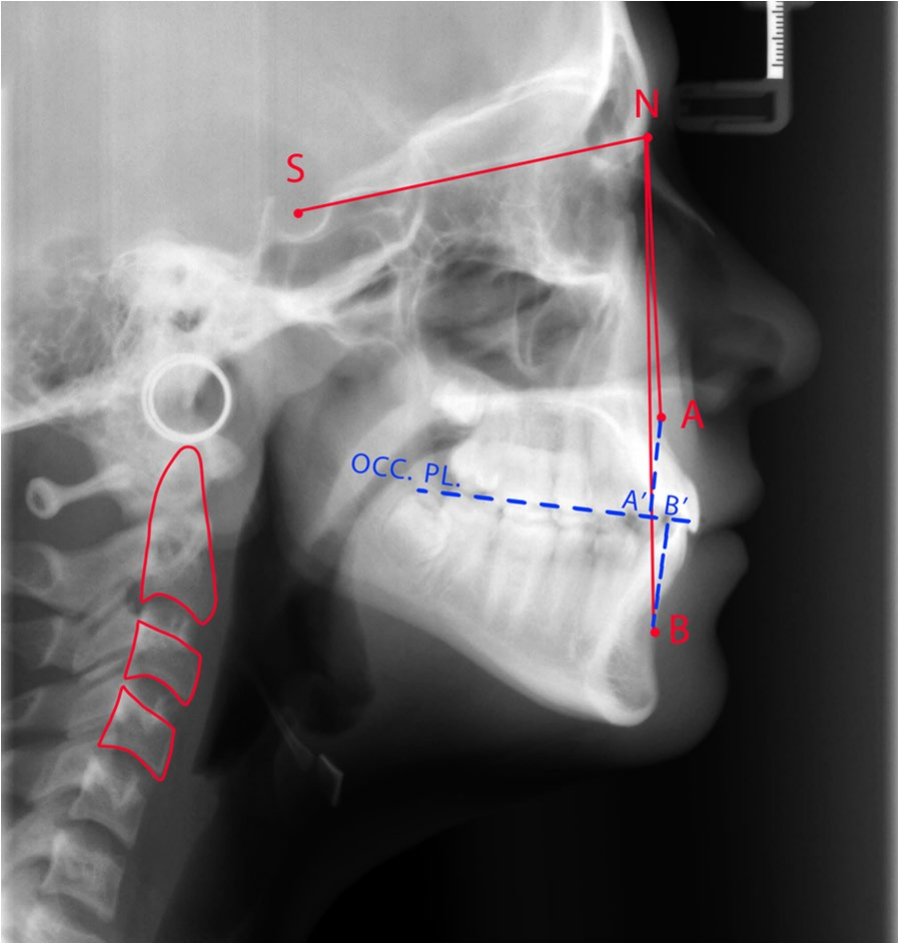
An example of cephalometric tracing, illustrating the CVM stage, ANB angle, and Wits appraisal. The C2, C3, and C4 vertebrae are outlined with red color. The concave inferior border of three vertebrae alongside the horizontal rectangular shape of C3 and C4 demonstrates the CS4 stage. Sella turcica and Nasion are represented by S and N, respectively. Point *A* displays the deepest point on the maxilla, between the anterior nasal spine and alveolus. Point *B* displays the deepest point on the curvature of symphysis [23]. Two lines perpendicular to *A* and *B* points are drawn, intersecting the occlusal plane (OCC. PL.) at *A*′ and *B*′. In this patient, the SNA and SNB angles were measured to be 81° and 79°, respectively. Consequently, the ANB angle was calculated to be 2°, representing a normal Class I relation. Moreover, the *A*′–*B*′ distance was equal to 2 mm, confirming the ANB angle

The six stages of CVM are described in Fig. 1 based on Baccetti’s method. If C2 and C3 had concave inferior borders, and C3 and C4 were trapezoids or horizontal rectangles, the subject was in CS3 (the onset of adolescent growth peak). If the inferior border of three vertebrae had concavity, and C3 and C4 were horizontal rectangles, the subject was in CS4 (the end of adolescent growth peak) [3]. Figure 2 demonstrates the method of tracing a lateral cephalogram. Considering mentioned criteria, 116 lateral cephalograms of class I and III subjects were selected, with equal distribution in two CVM stages (CS3 and CS4), and in two genders (Table 1).

**Table 1:** Descriptive information of sample population

Malocclusion classification (*n*)	Cervical vertebral stage	Chronological age (years)		Gender		Total (*n*)
		Minimum	Maximum	Female (*n*)	Male (*n*)	
Class I (68)	CS3	9	15	17	17	34
	CS4	11	16	17	17	34
Class III (48)	CS3	10	15	12	12	24
	CS4	11	16	12	12	24
				58	58	116

Cohen's kappa coefficient and Raw Agreement were used to assess intra- and inter-examiner reliability. The one-sample Kolmogorov–Smirnov test was used to investigate the normality of data distribution. The equality of variances was tested using Levene's test. The confidence interval was set at 95% in all tests, and *p* Values less than 0.05 were considered statistically significant. Due to normal distribution, the peak’s duration (CS3–CS4 interval) and the mean chronological ages of subjects at CS3 and CS4 were compared between class I and III subjects using independent samples *t* test. The same comparison was made between the two genders using independent samples *t* test. All analyses were done using SPSS Version 21 (IBM Corp., Armonk, NY) for Windows.

## Results

The intra- and inter-examiner agreements were greater than 90% and 82%, respectively, for CVM staging and skeletal relation assessment. The current study analyzed lateral cephalograms of 68 class I and 48 class III subjects (Table 1). The independent samples *T* test showed that the adolescent growth peak lasted 0.38 years (4.6 months) longer in class III subjects than in class I (*p* < 0.001). The comparison of mean chronological ages of subjects in the CS3 stage revealed that the difference between the two skeletal classes was insignificant (*p* = 0.630), implying that the onset age of adolescent growth peaks was similar in class I and class III (Table 2).

**Table 2:** Comparison of the mean duration of adolescent growth peak between two skeletal classes (independent samples *t* test)

Malocclusion classification	Mean chronological age (years ± SD)		Duration of adolescent growth peak (years ± SD)	Inter-class difference (years)	*p* value
	CS3	CS4			
Class I	11.91 ± 1.32	13.53 ± 1.33	1.62 ± 1.33	0.38	< 0.001
Class III	12.08 ± 1.31	14.08 ± 1.24	2.00 ± 1.27		

Also, independent samples *T* test showed that females experienced a 1.89-years adolescent growth peak on average while males experienced it for 1.69 years. The 0.20 years (2.4 months) difference between genders was statistically significant (*p* < 0.001) (Table 3). The adolescent growth peak initiated 1.44 years later (*p* < 0.001) and terminated 1.24 years later (*p* < 0.004) in male subjects (Table 4).

**Table 3:** Comparison of the mean duration of adolescent growth peak between two genders (independent samples *t* test)

Gender	Mean chronological age (years ± SD)		Duration of adolescent growth peak (years ± SD)	Inter-gender difference (years)	*p* value
	CS3	CS4			
Female	11.00 ± 1.04	12.89 ± 1.35	1.89 ± 1.19	0.20	< 0.001
Male	12.44 ± 1.10	14.13 ± 1.12	1.69 ± 1.11

**Table 4:** Comparison of the onset and termination age of adolescent growth peak between two genders (independent samples *t* test)

Cervical vertebral stage	Mean chronological age (years ± SD)		Mean difference (years)	*p* value
	Female	Male		
CS3	11.00 ± 1.04	12.44 ± 1.10	1.44	< 0.001
CS4	12.89 ± 1.35	14.13 ± 1.12	1.24	0.004

## Discussion

The CVM method, which is based on the morphology of three cervical vertebrae, is a common method of assessing skeletal maturation [3]. The adolescent growth peak is defined as the interval between CS3 and CS4, coinciding with maximum mandibular growth potential [2, 3]. The onset and duration of the mentioned period vary between skeletal classes, making the orthodontic intervention more challenging [18, 19, 21]. This study aimed to assess adolescent growth peak's onset age and duration in class III subjects compared to class I. Consequently, 116 lateral cephalograms of subjects from two skeletal classes were analyzed using the CVM method. Finally, the differences in onset age and duration of adolescent growth peak were assessed.

Multiple studies have confirmed the reliability of the CVM method for assessing skeletal maturation and predicting adolescent growth peak [14–16]. In contrast, few studies have called the correlation between CVM and skeletal maturity into question [11–13]. Santiago et al. found moderate to high reproducibility for the CVM method as well as low reliability in cross-sectional studies, warning researchers to use the CVM method cautiously [24]. Nestman et al. also resulted in less than 50% and 62% inter- and intra-examiner agreements, respectively [25]. The current study's inter- and intra-examiner agreements were over 82%, indicating high reproducibility. Rongo et al. stated that the higher orthodontic experience of a clinician does not necessarily enhance the reproducibility of the CVM method [26]. However, Khajah et al. found that examiner experience and training increased agreement level in CVM staging. Moreover, using 2D-lateral cephalograms resulted in the highest inter-examiner agreement, while 3D-CBCT images did not show any superiority over other types of radiography [27].

In the current study, the skeletal relationship was assessed using Steiner's analysis. Although this analysis is broadly used in orthodontic diagnosis [28, 29], using the ANB angle for defining skeletal relationship has been questioned due to potential misinterpretation and measurement errors [21].

The difference in the onset age of adolescent growth peak was not significant between class I and III subjects in the current study, indicating that this period begins at a similar age in both classes. Likewise, other studies have shown an insignificant difference in onset age between class I and III subjects [19, 20]. For instance, Qureshi et al. resulted in a similar mean onset age of adolescent growth peak for all subjects of three different classes [30]. In contrast, few studies have supported the theory of delayed adolescent peak in class III subjects compared to class I [18, 19]. This variety in results may come from multiple factors that affect the onset and duration of growth peak, including gender, ethnicity, nutritional habits, genetics, environment, and lifestyle [4–6].

Based on our findings, the mean duration of the adolescent growth peak was 1.62 years in class I subjects and 2.00 years in class III subjects. This 0.38 years (4.6 months) difference was statistically significant. Kuc-Michalska et al. reported that the duration of adolescent growth peak was 11 months and 16 months in class I and class III Caucasian subjects, respectively. Moreover, this 5 months difference was statistically significant [19]. Other studies also reported a more extended adolescent growth peak in class III subjects compared to class I, with a difference of 4.8 and 5.9 months [20, 21]. Considering subjects’ craniofacial growth component in sagittal and vertical planes, Szemraj-Folmer et al. concluded that class III and class I patients with skeletal open bite experience a significantly longer adolescent growth spurt compared to class I patients without open bite [31]. Our findings confirm that their longer adolescent growth peak may explain class III subjects' greater mandibular growth. As a result, class III patients may require longer use of orthopedic devices until their adolescent growth peak ends [21]. However, additional longitudinal studies are required to investigate this hypothesis.

As reported, females had an earlier and longer adolescent growth peak than males. According to Jeelani et al., the adolescent growth peak began 4 months earlier and lasted 1.5 months longer in females [21]. In another study, Qureshi et al. concluded that class III boys experience a later onset and also a later termination of the adolescent growth peak. Moreover, it is stated that the shortest and the longest growth peak occurs in class II and class III boys, respectively [30]. Confirming the findings of above-mentioned studies, Arriola-Guillen et al. stated that the adolescent growth spurt occurred one CVM stage earlier in women compared to men [32]. However, there are studies that have denied any significant difference in the duration of adolescent growth peak between genders [19, 33]. Based on probable differences between males and females, it is suggested considering patient’s gender when deciding the timing of an orthodontic treatment.

Retrospective cross-sectional studies may not truly represent longitudinal changes. However, we tried to intensify the significance and validity of our results by reaching the required sample population in each group. Moreover, the limited sample size did not allow us to compare subjects’ malocclusion class and gender simultaneously. To obtain more reliable outcomes, further longitudinal studies are needed to track skeletal changes over time. However, requiring extra exposure to X-rays makes this approach somehow unethical [21]. Another limitation of the current study was that patients' chronological ages were recorded by years in the archive. To obtain more accurate results, it is recommended recording the chronological age by month.

## Conclusions

- The mean onset age of adolescent growth peak was the same in both class I and III subjects.
- The adolescent growth peak lasted 0.38 years (4.6 months) longer in skeletal class III subjects than in class I subjects.
- The adolescent growth peak initiated earlier and lasted longer in female subjects.

## Data Availability

The datasets used and/or analyzed during the current study are available from the corresponding author on reasonable request.

## Abbreviation

CVM: Cervical vertebral maturation
